# Philosophical Progress
*The Biographical History of Philosophy, from its Origin in Greece down to the Present Day. By George Henry Lewes. Library Edition, much enlarged and thoroughly revised. London: John W. Parker and Son, West Strand. 1857.


**Published:** 1857-10-01

**Authors:** 


### Art. IV.—PHILOSOPHICAL PROGRESS.*

Mr. Lewes is an accomplished man and a brilliant writer. The charm of his style always makes a pleasing subject still more pleasing to his readers. But while we admire the great talent with which he writes, the clearness with which he expounds, and the vigour with which he argues, we are unable to keep on friendly terms with him for many consecutive pages.

Mr. Lewes is a determined unbeliever in philosophy, and points to its unprogressive character as evidence of its aiming at impossibilities. Every attempt of the giant intellects of the past to scale the heaven of ontology by heaping mountain upon mountain, has been defeated by some redoubtable Hercules mighty in the art of destruction; and so it will ever be, Mr. Lewes would say, when mortals presume to arrogate the prerogative of gods. But in an age when mortals have surpassed the divinities of the ancient world—in an age when many alleged impossibilities have been accomplished, and many wonders have become common things in less than a quarter of a century, we, with good reason, prefer regarding the circular movement which our author ascribes to philosophy as a spiral movement, forced indeed to return towards the point from which it originates, but always receding from it in some slight degree, and always tending to an apex, from which, with great force, it will eventually start in a direct line to form the science of sciences.

Now, it seems to us that philosophy has described a circular course, not because it attempts impossibilities, but because on each departure from nature it had not made its basis firm and ample by rigid induction. In consequence of this defective beginning, scepticism has invariably overthrown its conclusions; this necessitated a fresh return to nature, when a new foundation would be laid, free from the most obvious defects of the preceding one, but still faulty, and destined again to be more carefully examined and improved. The repetition of this course again and again amounts, as Mr. Lewes thinks, to the condemnation of philosophy. But had not astronomy and chemistry to describe a devious course in the wilderness of empiricism before they reached the promised land of science?

"If no attempts were made to draw a conclusion, and see what use could be made of it, till *grounds formally complete* were before us, conclusions would never be drawn. The certainties by which the chemist, the astronomer, the geologist, conducts his operations with

* The Biographical History of Philosophy, from its Origin in Greece down to the Present Day. By George Henry Lewes. Library Edition, much enlarged and thoroughly revised. London: John W. Parker and Son, West Strand. 1857.

composure and success, were once bare possibilities, which after being handed back and forward, between Induction and Deduction, turned out to be truths."*

Is not this just what has been taking place in philosophy? Absurd and monstrous conclusions have invariably driven men back to nature, to repeat the inductive process, and their success has been commensurate with the knowledge they possessed, or gained, of the laws of inductive reasoning; deduction being sure to develope any imperfection in such knowledge.

If we regard scepticism, to use an anatomical term, as the *pylorus* that will not allow crude and indigested matter to enter the temple of science—as the disposition to admit no proposition to be true without the assent of reason—we must confess that, so far as scepticism has been satisfied, philosophy has described a linear movement. Even Mr. Lewes himself chronicles the fact that from Pyrrho to the New Academy some advance had been made, but without intending to do so, we imagine. "Ethics had become elevated to the rank of a science." (?)† Every fresh return to the fountain-head of nature, after scepticism had destroyed the incredible systems of philosophers, was a sort of moral recoil from the offensive conclusion that absolute certainty was unattainable. Socrates and Reid, each in a remarkable manner, initiated a reactionary movement of this moral character.

From the New Academy to Hume the linear movement has been considerable. While the Pyrrhonists and the New Academy proclaimed scepticism as the final result of all inquiry, Hume only doubted the existence of noumena—phenomena were beyond the reach of his scepticism. He says: "Whoever has taken the pains to refute the cavils of this total scepticism has really disputed without an antagonist."‡ Here, then, is some ground gained. Whether it has been *so positively* secured as is commonly believed, remains to be seen. For instance, what are we to think of Reid's repeated attempts to convince his readers that Hume was inconsistent in being only half a sceptic. Thus in one place he says:—

"The author of 'The Treatise of Human Nature' appears to me to be but half a sceptic. He hath not followed his principles so far as they lead him; but after having with unparalleled intrepidity and success, combated vulgar prejudices, when he had but one blow to strike, his courage fails him, he fairly lays down his arms, and yields himself a captive to the most common of all vulgar prejudices—I mean the belief of the existence of his own impressions and ideas."§

Sir William Hamilton is at variance with Reid on this point:

* Outlines of the Law of Thought, by W. Thompson; p. 313.
† Page 284. ‡ Human Nature, p. 250.
§ Inquiry, Hamilton's Edition, p. 129.

"In Reid's strictures upon Hume," he says, "he confounds two opposite things. He reproaches that philosopher with inconsequence in holding to 'the belief of the existence of his own impressions and ideas.' Now if, by *the existence of impressions and ideas*, Reid meant their existence as mere phenomena of consciousness, his criticism is inept; for a disbelief of their existence, as such phenomena, would have been a suicidal act in the sceptic."*

In Note A,† Sir William Hamilton enters more fully into this distinction. There he declares himself to be a demonstrator in relation to the facts of consciousness, but in relation to the veracity of consciousness, a dogmatist. Now, Reid was a dogmatist in both respects, and he could not see what ground there was for doubting the existence of external objects, any more than the existence of consciousness and its phenomena. Referring to the fact of Hume's not calling into question the existence of impressions and ideas, he asserts :—"I am persuaded that there is no principle of his philosophy that obliged him to make this concession."‡ This is a confusion and inaccuracy, according to Hamilton. Mr. Lewes takes virtually the same view of this point, and evidently entertains a very mean opinion of Reid's penetration. Now, as far as we have been able to perceive, the common-sense philosopher has more truth on his side than his more demonstratory critic ; our reason for making this assertion we shall here append.

Every perception has two elements—a subjective and an objective—the object perceived, and the apprehension of that object. Now, there is a perception which enables us to become *speculatively* assured of the trustworthiness of perceptive acts in general. The objective element of this perception is, the primariness of the veracity of consciousness—the subjective element, the apprehension of this fact. In *the order of nature* the objective element is first. In *the order of knowledge*, the subjective. That is, the veracity of consciousness exists as a fundamental fact before we can possibly be aware of it; but, on the other hand, till we become thoroughly aware of it, that is, till we acquire the perception we have just mentioned, it is not only possible, but unavoidable that reason (to which faculty we are indebted for the perception) should be undecided on this point.§

* Inquiry, Hamilton's Edition, p. 129, note.

† Hamilton's Edition of Reid's Works, p. 742.

‡ Inquiry, &c. p. 130.

§ It is necessary to be precise on this head, because there are those who will argue that reason must start from data which do not admit of being proved, being self-evident; and that consequently there is a source of truth, the veracity of which does not allow of demonstration, because all reasoning must suppose it veracious; but that is practically, and as a matter of faith. But the data from which reason draws conclusions are not necessary and universal propositions: all such propositions, as we shall show in the sequel, are inferred from elementary facts. Now the statement that the veracity of consciousness is the fundamental fact is necessary and universal, it is not immediately perceived, but is acquired by reason. Although, then, the *knowledge* that the veracity of consciousness is the fundamental

But once this knowledge is gained, reason is forced to be acquiescent for ever afterwards. For let it after this endeavour to prove the veracity of consciousness—that having been demonstrated to be the fundamental fact of consciousness—a *petitio principii* excludes any such attempt; or let it try to *disprove* it, and it finds the disproof to be impossible, for a subversion of the *principium* would be the consequence.

Now this line of argument, though bearing a strong similarity to that carried on by Reid and Stewart, differs from it in this:—Their argument was, that the veracity of consciousness being a fundamental fact, it must be accepted as a self-evident truth. Ours is, that *reason* perceives that it is a fundamental fact, and that it consequently cannot be proved or disproved. They were pure dogmatists. We endeavour to be pure demonstrators. We believe, as they did, that consciousness is equally credible in all its deliverances. But we believe, differently from them, that since reason seeks to be satisfied on this head, it ought to be supplied with its own special evidence, instead of being silenced by the taunts and vociferations of dogmatism.

Sir William Hamilton holds a position midway between these two. He holds that if we doubt the existence of consciousness and its phenomena, the doubt refutes itself; but that if we doubt the truth of the testimony of consciousness to aught beyond its own ideal existence, it does not refute itself. The principle which we have stated above, warrants us in asserting that it does. Let us enter more fully into Hamilton's reasoning on this subject.

He lays great stress on the fact that the data or deliverances of consciousness, considered simply in themselves as apprehended facts or actual manifestations, are above all scepticism:—

"For as doubt is itself only a manifestation of consciousness, it is impossible to doubt that what consciousness manifests—it does manifest, without in thus doubting, doubting that we actually doubt, that is, without the doubt contradicting and therefore annihilating itself."*

Now, this argument leaves the vital point still open to the assaults of scepticism. Your argument is valid, the sceptic might say, but it evidently involves a principle which, if unsound, causes it to partake of all the imperfections of a vitiated source: that principle is the veracity of the faculties which afford you fact is the primary fact *for us*—this knowledge is not obtained by means of a *primary* cognition. The veracity of consciousness does not exist *for us* as a certainty till it becomes the objective element of the perception in which it is revealed to us, and of every perception the subjective element is first in the order of knowledge, while the objective is first in the order of existence. Vide this Journal for Jan. 1857, Art. II., On the New Scottish Philosophy.

* Note A, p. 744.

your argument. Simply so far as it is a trustworthy discloser of objects can consciousness be a voucher, not only for the existence of aught distinct from itself, but even for its *own* existence. Is consciousness trustworthy?

"A mere disposition to believe, even if supposed instinctive, is no guarantee for the truth of the thing believed. If indeed the belief ever amounted to an irresistible necessity, there would then be no *use* in appealing from it, because there would be no possibility of altering it. But even then the truth of the belief would not follow; it would only follow that mankind were under a permanent necessity of believing what possibly might not be true; just as they were under a temporary necessity (quite as irresistible while it lasted) of believing that the heavens moved and the earth stood still."*

When the sceptic asks, therefore, is consciousness a faithful guarantee for the truth of all its declarations? it is no answer to his question to insist upon the impossibility of altering some of our beliefs, even when this impossibility is of an absolute, and not of an accidental nature. It is impossible to be cognizant of an object without believing in its existence; to be conscious of an object, and to believe in the existence of that object, are inseparable acts. "All consciousness is realised in the enunciation—That is there (or this is here)."†

"If we attend," says Reid, "to that act of our mind which we call the perception of an external object of sense, we shall find in it these three things:—*First*, some conception or notion of the object perceived; *secondly*, a strong and irresistible conviction and belief of its present existence; and, *thirdly*, that this conviction and belief are immediate, and not the effect of reasoning."‡

Abstract from an act of consciousness belief in the existence of its object, therefore, and you destroy it as effectually as you would destroy water by taking away its oxygen. This accounts for the impossibility of practical scepticism. Try your utmost to believe yourself non-existent—make every effort you can to believe that a tree is part of your own being, and you never can succeed, for success in such an attempt would be the annihilation of the sane mind.§ This is primitive belief, arising merely from the presentation of the object to the immediate perceptive power of the mind; that is, it does not depend on any previous act of thought, but is itself an element essential to that act of consciousness which every other supposes. Now, human thought

* Mill's System of Logic, Third Ed. vol. ii. p. 95.

† Sir William Hamilton, Note D*, p. 878.

‡ Essays on the Intellectual Powers, Hamilton's Ed. p. 258.

§ "Nature," says Hume, "by an absolute and uncontrollable necessity, has determined us to judge as well as to breathe and feel; nor can we any more forbear viewing certain objects in a stronger and fuller light upon account of their customary connexion with a present impression, than we can hinder ourselves from thinking as long as we are awake, or seeing the surrounding bodies when we turn our eyes towards them in broad sunshine."—*Human Nature*, p. 250.

is composed of many beliefs besides these, but none of them is fundamental; as *mere* beliefs or opinions, their negation is quite conceivable, because it does not involve, as in the case of our primitive beliefs, the annihilation of all consciousness. They rest on evidence, and, unless the evidence is conclusive,* it matters not how intense or persistent they be, they are all liable to be proved erroneous. From not seeing the distinction which exists between primitive belief, and belief resting on incomplete evidence, no less a writer than Mr. J. S. Mill has been led into error and confusion on this subject.

"There is no proposition of which it can be asserted that every human mind must eternally and irrevocably believe it. Many of the propositions of which this is most confidently asserted great numbers of human beings have disbelieved. The things which it has been supposed that nobody could possibly help believing, are innumerable; but no two generations would make out the same catalogue of them. One nation or age believes implicitly what to another seems incredible and inconceivable; one individual has not a vestige of a belief which another holds to be inherent in humanity. There is not one of these supposed instinctive beliefs which is really universal."†

Now, does Mr. Mill mean to maintain that there are no instinctive beliefs whatever? Scarcely. In charging the enemy he has gone too far, and exposed himself in flank and rear. Because many of the beliefs which men held to be instinctive were afterwards proved to be not universal, it does not follow that there are no beliefs co-extensive with human thought in every age of the world. Wherever a human mind exists, there exists perception, and of perception belief in the present existence of an object is an inseparable element.

But although we are so constituted that it is impossible for us to have a perception without our having, too, as one of its elements, a conviction that its object exists as it appears to us to exist, there is nevertheless something in us which desires satisfaction on this point, such as instinctive belief does not afford it; and whenever this something becomes active in a man's mind, although he is practically constrained to act and think as the sane portion of mankind, and would confess himself demented were he conscious of doing otherwise, still speculatively—in that region of the mind which judges from the comparison of propositions, that is, through evidence—he will be all in amaze, wondering if it be possible ever to discover a reason for the faith that is in him. Now this is philosophical scepticism, and is the furthest extent to which doubt can reach while the mind

* When evidence is conclusive—when a proposition is *proved*—practical scepticism is also impossible in regard to it. The only doubt then admissible is that touching the veracity of the faculties which declare that a proposition must be true when it is proved.

† Mill's System of Logic, vol. ii. &c. p. 36.

retains its sanity. It arises from reason's* having a strong desire to be possessed of its special evidence—evidence which instinctive belief does not afford it; and until this is supplied the doubt cannot be suppressed, especially when, as it was in the time of Hume, "we are necessitated by reasoning to contradict the primary instincts of nature, and to embrace a new system with regard to the evidence of our senses;" and no dogmatism, however well grounded and however ably and forcibly expressed, suffices to quiet reason's restless longings. To call such a spirit of inquiry metaphysical lunacy, as Reid did, evinces that he had confounded a possible doubt with an impossible—the doubt of réason in search of its proper evidence with the withdrawal of that fundamental belief which inseparably accompanies an immediate, and indeed a mediate perception, which is an impossibility—an impossibility which proves that all the ridiculous actions attributed to Pyrrho were fabrications of an opponent who was illogical in his conclusions. A man is not impelled to commit all sorts of ridiculous actions, because *speculatively* he cannot satisfy his mind concerning the integrity of consciousness, and does not choose to live *in this respect* a life of faith—except it be provisionally.

But while speculative doubt does not involve practical doubt, it is quite as possible to be sceptical in regard to the *facts* of consciousness as it is to be regarding the truthfulness of its testimony; for the argument which Sir William Hamilton, and according to him, Cousin, look upon as placing the phenomena of consciousness high above the reach of scepticism, can only be regarded as decisive, so far forth as the faculties from which it proceeds are trustworthy. As long therefore as the principle of veracity receives nought but dogmatic support, so long will it be legitimate for the restless reason of man to ask—Do my faculties deceive me, or do they not? Since, then, the principle of veracity underlies even the existence of consciousness itself, it is manifest that the phenomena of consciousness are not a whit less open to question than the truthfulness of the same when it attests the existence of external objects. If Reid erred, therefore, in making no distinction between speculative and practical doubt, he nevertheless saw more clearly than his critic seems to have done, that both the facts and the testimony of consciousness involved, in an equal degree, the principle of veracity; that if the one were doubted, so ought the other; but that if the one were taken on trust, so ought the other. Thus in his strictures on Descartes, he asks why that philosopher did not prove

* We believe that we are amply justified in rejecting the distinction that has been made since Reid's time between reason and reasoning. Reasoning we consider to be the operation of the faculty of reason.

the existence of his thought. "Consciousness, it may be said, vouches that. But who is voucher for consciousness? Can any man prove that his consciousness may not deceive him?"* And again, when criticising Hume, he asks,—

"What is there in impressions and ideas so formidable, that this all conquering philosophy, after triumphing over every other existence, should pay homage to them? Besides, the concession is dangerous; for belief is of such a nature that, if you leave any root, it will spread, &c. A thorough and consistent sceptic will never, therefore, yield this point; and while he holds it, you can never oblige him to yield anything else. To such a sceptic I have nothing to say; but of the semi-sceptics I should beg to know, why they believe the existence of their impressions and ideas. The true reason I take to be, because they cannot help it; and the same reason will lead them to believe many other things."†

Reid, then, had more than an obscure view of the principle of veracity; he saw it clearly as a dogmatist, but would not see it, nor permit any one else to see it, in any other way. He assayed to erect dogmatism into a final instead of a provisional system, which is an insult to reason.

We believe that Sir William Hamilton is wrong, then, in his estimate of the difference between rational and irrational scepticism, and in his criticism on Reid's mistake in this respect; and also when he thinks that the facts of consciousness are radically less open to question than the testimony of the same, seeing that the principle of veracity underlies both, seeing that in this case we have no stronger evidence for the existence of the traveller than that he declares positively, and to the satisfaction of reason, that he does *exist*, which is the same evidence that we have for the truth of what he *narrates*.

The tenor of our remarks is, that scepticism has yet a reserve to bring up against its elated opponents, and an invincible one. But we are not sceptics without also being dogmatists, and we are neither one nor the other by choice; what we aim at is demonstration; we desire to satisfy the legitimate demands of reason.‡ As sceptics, we think sensationalism has yet to re-examine its foundation; as dogmatists, we think that most of the

* Inquiry, &c. p. 100. † Ibid. p. 130.

‡ When these are satisfied, and not before, we shall be in possession of the long and passionately desired Criterium of Truth. It is reason alone that asks for this Criterium, and when reason has obtained it, we may rest assured there will no such absurd question be repeated as that which Mr. Lewes puts in the mouth of the ancient Sceptics:—"Very well," reply the Sceptics; "Reason is your Criterium. But what proof have you that this Criterium itself distinguishes truly? You must not return to sense, that has been already given up, you must rely upon reason; and we ask you what proof have you that your reason never errs?—what proof have you that it is *ever* correct? A Criterium is wanted for your Criterium; and so on *ad infinitum*." (p. 227.) The obvious answer to this objection is, that when reason is supplied with its special evidence it is convinced, and becomes acquiescent. To ask for proof that reason, when supplied with proof of the veracity of consciousness, decides truly, is to suppose reason behind reason, of which we have no evidence.

mighty and all-absorbing questions of all the past are yet in the womb of dogmatism, which is the real pioneer of intellectual and moral progress, and at the same time the great conservative power that has upheld many a dominant principle which sensationalism attempted to refute and condemn, and that such questions demand for their solution a more advanced method than the "positive,"—than that which has no place for universals, and pronounces essences and causes to be unknowable and problematical entities.

Having now characterized what seems to us the real state of philosophy at the present moment, we are able to point out more clearly than we otherwise should have been, the position which Mr. Lewes and the school he represents hold among the different systems which are in repute.

Sensationalism, for we must so term a system which has no recognised place for the superior faculties, is, as to phenomena, demonstratory: as to the disclosures of consciousness touching aught beyond the limits of the *ego*—sceptical. Now, in this its sceptical phase, it manifests a spirit of finality very unwise, and a contractedness certainly pernicious. Scepticism, to be of any value, must be negative demonstration. Prove that essences and causes cannot be known, that universals are unattainable, and we bow at once to such a decision. You think you *do* prove all this, but what is it you ground your conclusions upon? The circular movement of philosophy; the impossibility of sensations existing out of sentient beings—the impossibility of obtaining ideas from any other sources than sensation and reflection. Now, as to the first objection, it amounts to the fallacy of limiting what *will* be to what *has* been, and would not, we believe, have been urged had it not been deducible from the supposed impossibility of philosophy. As to the second, no one ever dreams that a sensation can have any existence out of a sentient being, but consciousness declares that certain of the objects which it apprehends are not sensations. For instance, the primary qualities of bodies are as different from feeling of any kind as it is possible to conceive anything to be. Indeed light and sound are not feelings, otherwise they could not be apprehended *as* distant objects. The very perfection of the organs of sight and hearing is that they cause us to be aware of the existence of distant objects by means of manifestations apprehended *as out* of our organism, and in the case of sight *as* possessing a degree of extension vastly exceeding that of the whole organic unity. How, then, can we regard visible objects as feelings, or affections of the organism as animated, when to be apprehended *as external* to that organism,* and as extended over a surface vastly greater

* The mere visible object, *e.g.*, considered aloof from the inferences connected with it, is apprehended *as out* of the organism. That which in the case of sight is first in the order of knowledge is cognised *as* external, and if it were not, if it were than its own is felt to be, is contrary to all we know of feeling? We consider, therefore, that consciousness reveals three main classes of objects.

1st. Internal objects, modifications of self as animated.

2nd. Internal objects, not felt, but apprehended *as external*, and only inferred to be internal from comparing together the primary data of such objects.

3rd. External objects, apprehended as external, and which, when their primary data are compared, cannot be inferred to be aught else; and to deny the externality of which amounts to a denial of the veracity of consciousness.

Here then is one class of objects which we cannot bring ourselves, either by effort or by argument, to regard as sensations, modifications of consciousness, or anything of that kind. That when we cognise tangible qualities we are also conscious of a sensation in the nerves of touch we freely confess, but this most assuredly is not all that we are conscious of;* the sensation is accompanied with an object which is not a sensation—not a modification of the feeling, or of the thinking subject; so consciousness declares. And since consciousness knows itself, it knows that the object is not a part of itself; and since it knows all manifestations of feeling, *per se*, it knows that the object is not like them, and consequently pronounces it (from a knowledge of it, *per se*, and from a knowledge of all internal objects, *per se*) to be a *non ego*.

Now, to deny that consciousness is veracious in this deliverance involves us in inextricable confusion. We imply thereby that it cannot discriminate its objects accurately, that it pronounces some of them to be out of us when they are really within us; and if it cannot distinguish between object and object, it may not be capable of distinguishing between itself and its objects; so that reason becomes tossed to and fro upon a stormy sea of doubt without chart and compass. Now, out of this confusion the only way to escape is to become dogmatists, and believe "that we are (not) created capable of intelligence, in order to be made the victims of delusion;" "that God is (not) a deceiver," "and the root of our nature (not) a lie;" or if dissatisfied with mere belief on this point, we must endeavour to procure such evidence as will convince reason that consciousness is not mendacious.

cognised as a feeling, how could we regard it *as out* of the *ego* by means of an association or an inference? A feeling *out* of the sentient unity! It is a contradiction in terms. Is it supposed that colour is literally a sensation, like pain, in the eyeball, and that we associate an external object with it? In that case it would be impossible for us to cognise a visual object *as* occupying the same place as that object considered as tangible, which we clearly do.

* Indeed this sensation is rarely attended to, attention being fixed upon the object, not upon the sensation.

As to the third head, the impossibility of obtaining propositions rigidly universal, because experience is finite, we must beg the reader to wait till we get further on.

Now, although we condemn the sensationalists for their contracted and unbelieving spirit, our opposition to them is not of an *a priori*, but an *a posteriori* nature; starting from the same point, and following the same direction, we believe that the road does not close where they so loudly protest that it does. That the sensational method is the true one, so far as it goes, let the grand and rapid march of the physical sciences proclaim. But the time has evidently arrived when the extension of that method, so as to gain the adherence of more idealistic thinkers has become a great social necessity. The philosophical dogmatists, no less than those who have hit upon processes of investigation without perceiving them to be laws demanding to be expressed in general terms, have undoubtedly prepared material, which it now becomes the duty of the demonstrators to test and define, in order to make the true method adequate to the moral and social requirements of this, in these respects, doubting and unsettled age. And it is only by taking possession of the material thus waiting for their appropriation that they can accomplish so desirable an end.

Now the course for the demonstrators to follow is to abstain from the suicidal act of doubting the truth of the testimony, while they hold the facts of consciousness, by which they lay themselves open to defeat from scepticism; and to satisfy the legitimate demands of reason as to the truthfulness of our faculties, in order to do which we must have recourse to that operation of the mind which infers that certain truths must be universal. Now, such a power is denied the mind by the sensationalists; they incapacitate themselves, therefore, for securing their ground against scepticism. To the task of describing how we perceive that certain propositions must be universal, we now address ourselves.

Mr. J. S. Mill defines reasoning, in the fullest sense, to be inference from particulars to particulars. "All inference," he writes, "is from particulars to particulars. General propositions are merely registers of such inferences already made, and short formulæ for making more."* But how can we conclude from particulars to particulars with absolute certainty? That all rigid deduction is from universals we cannot for a moment doubt. The *dictum de omni et nullo* we consider to be as firmly grounded as it is possible for a principle to be. How can you know that your particular applies to every case that you may have occasion to bring under it? You cannot; for then it

* System of Logic, &c. vol. i. p. 216.

would be no longer a particular, but a universal, a proposition that did not admit of an exception; and until you know that it applies to every possible case, you can only draw probable conclusions from it. This truth will become so evident, we trust, as we proceed, that it becomes unnecessary in this place to dwell any longer upon it; let us proceed, then.

If we observe but a single fact we cannot know that another is connected with it, except it be connected with it in the mind. It is by means of a mental *nexus*, therefore, that we know that one thing is connected with another when only one of them is observed. And now this *nexus* must be either a universal, or a particular proposition; or a proposition the quantity of which is indetermined. If it be the first, we infer without hesitation, that the unobserved fact is connected with the observed; if it be the second, we infer that it may or may not be; if it be the third, we infer that the unobserved fact is connected with the observed, but whether necessarily or contingently we cannot tell. As far as experience extends, one fact may be invariably joined to another, as that the feet of all horned animals are cloven, and we may deduce from such a proposition, and perhaps never find ourselves wrong; but evidently uniform experience—*inductio per enumerationem simplicem*—does not enable us to decide whether the conjunction between two facts be necessary, or whether contingent merely, but uniform, as when two distinct phenomena are invariably found together, but are both effects of some known cause or joint causes. Thus there is an invariable connexion between the angles of a triangle, but no one angle is the cause of another angle.

Again, *two* facts being observed joined together, we have but an *indefinite* notion of the nature of the junction which exists between them, unless it happen that we *know* the nature of it previously. This previous knowledge is the mental *nexus* by means of which we determine the nature of the junction which subsists between the two facts. If the *nexus* is a universal, the junction between the two facts is necessary; if merely a contingent proposition, their junction is contingent; if an indefinite proposition, their junction is indefinite. To know in this manner by means of a mental *nexus* is deduction; and the only rigid deduction, such, for instance, as science demands, is that which proceeds from universals.

To illustrate this process of reasoning more fully, let us quote the following passage from the chapter on Reason in Locke's Essay, and briefly comment upon it.

"Tell a country gentlewoman that the wind is south-west, and the weather louring and like to rain, and she will easily understand it is not safe for her to go abroad thin clad in such a day, after a fever: she clearly sees the probable connexion of all these—viz., south-west wind, and clouds, rain, wetting, taking cold, relapse, and danger of death, without tying them together in those artificial and cumbersome fetters of several syllogisms that clog and hinder the mind, which proceeds from one part to another quicker and clearer without them."*

Now, without agreeing with Locke in his wholesale condemnation of the syllogism, we think that the mind perceives the connexion between one of these terms and the next to it by deductive reasoning, and that consequently there is a simpler form of ratiocination than that to which the syllogism gives expression. The fact of the gentlewoman's uniting each of these facts with the one immediately following, proves that she thought of them as so connected; that is, each pair of terms, the first and second, the second and third, &c., came as a subaltern under its containing proposition in her mind; and according as the containing proposition was universal, contingent (mostly or commonly true); or indefinite, so would her conclusion be necessary, contingent, or indefinite in the junction of its terms. It rains, some one tells me: rain is contained in the proposition: all rain wets: therefore I conclude that this rain wets, though I do not observe it. The whole process when expressed assumes this form.

1st. Where only one fact is observed or stated.

It rains,
All rain wets,
Therefore, *this* rain wets.

2nd. Where two facts are observed or stated to be joined, but the senses are not able to determine the nature of their conjunction.

The cow ruminates,
All cows must ruminate,
Therefore, *this* cow *must*.

The first premise suggests the second—the second enables us to infer the third.

Now, the ordinary opinion is, that deductive reasoning consists primarily in proving that one term is connected with another by means of a middle term, which must be distributed in one of the premises at least.† Thus in *homo, animal, vivens—homo* is connected with *vivens* by means of *animal*, as in a chain of three links, the first is united to the third by means of the second; consequently the syllogism proves *mediate* junction between the two terms of the conclusion. This junction is enough to exonerate it from the charge of inutility so frequently brought against it; for mediate junction, if it has to be *deduced*, must be deduced according to the syllogism implied or expressed. We

* Essay, b. iv. c. xvii. sect. iv. p. 513.

† We omit the mention of arguments with a negative premise for the sake of brevity.

think, then, that the *first* step of deductive reasoning is that given by us above; and that the syllogism is the *second* step, that it is, in fact, the first step of the Sorites.

Having shown that the simplest form of ratiocination is determining the necessary, contingent, or indefinite—*immediate* connexion of facts by means of universal, contingent, or indefinite propositions, we have next to point out how these are obtained.

Necessity and contingency are related notions, the first being the positive, the other the negative. The only idea of conjunction derivable from simple observation is what must be called indefinite or undetermined. Unvarying experience can only afford a strong presumption that two facts are necessarily connected: yet association has frequently fastened the connexion between two facts which have been invariably observed together, or between two ideas which have been always thought of together, so firmly that it was found impossible to undo it when it was afterwards proved contingent. In consequence of the difficulty experienced in destroying a long-standing association, some believe that the notion of necessity can be thus accounted for. If our experience all tends in one direction, and affords us no model or analogy to facilitate our conceiving two facts apart, then say they, we find it necessary to think of such facts always as they are presented to our experience, and the only necessity in the connexion of facts is this. Now, what clearly proves the erroneousness of this view is, first, that invariable co-existence or succession of phenomena, and association arising therefrom, are not always present where the notion of necessity is caused. One instance of what is required to prove necessary connexion does as well as a million; secondly, the notion of necessary conjunction is caused where there *are* facilities afforded of framing a different notion, where there are *not wanting* analogies or models to assist us in imagining the two facts apart, and where it is indispensable to ascertain that such a conception of them is absolutely excluded.*

* The man who is said to have seen a French baby with a long nose (page 558 of our author's work) was under no necessity of thinking of all French babies under this type for ever afterwards. Yet it is possible that by continuing for a long period without attempting to think of them as differing from this specimen, an inveterate association might grow up in his mind between French babies and long noses. But not necessarily. The thought ought naturally to occur to a thinking mind that some French babies had short noses. For it is only when the supposition of a different combination of facts (such a supposition in the majority of instances being easily framed and readily suggested) is excluded by our knowledge that we are forced always to think of the unknown as precisely similar to the known. The ancients might have thought that there were black swans—for the connexion between swans and whiteness being to them a mere indefinite one, it did not exclude the conjecture that swans might vary in colour. Even where we have no means of imagining an object different from the model we possess of it, we can only say, we cannot picture it otherwise—not that it cannot *exist* otherwise, which is the character of a necessary connexion: when we *perceive* that the supposition of its being different is incompatible with our knowledge of it, then it is that we conclude that it cannot *exist* otherwise.

It is quite possible to conceive that oxygen and nitrogen might unite in other than definite proportions to form air till the supposition is precluded by accurate knowledge of its nature.

Now let us proceed to show that the notion of necessity enters as largely into the composition of thought as the commonest facts of mere sense-apprehension; for sense-apprehension is always attended with an inference of the following kind. When, for example, we see a stone, the mind runs through the form of deductive reasoning given above, implicitly and instantaneously. I see a stone: all stones are hard: therefore this stone is hard; and now this inference is not conclusive unless hardness and stones are necessarily connected in the mind. But let us state another example better calculated to lead us towards the point at which we are aiming. The statue is placed on a pedestal: all that is evident here to the senses is the relation in place which the statue and the pedestal bear to each other. But we assert with undoubting confidence that the statue *depends* upon the pedestal for retaining the place it does. How do we obtain this notion of dependency? By deducing it from a universal. But how was this universal first obtained? The relation which the two objects bear to each other in place certainly does not afford it. Let us search for it then in some other quarter. We observe that if an object placed under another, as a pedestal under a statue, is removed, the upper falls. Does this fact alone afford us the notion of dependency? No: for here all that is obvious to the senses is that one event is followed by another—mere antecedence and consequence—which Hume very justly maintained cannot originate the notion of a causal nexus. And which Reid clearly proved to be quite incapable of begetting the idea of necessity, seeing that then day must be considered as the cause of night, or night of day.

Mr. J. S. Mill endeavours to rescue the sensational theory of causation, thus overthrown, by adding that invariable sequence is not synonymous with causation, unless the sequence, besides being invariable, is *unconditional*. But here the word unconditional is intended to express more than the advocates of the sensational theory have a right to, exhibiting how difficult it is to keep within prescribed bounds, when those bounds are an arbitrary limit to the real powers of the mind, and how those who deny any of the faculties are forced to imply the very faculties they deny;—or it expresses merely one of the facts connected with causation, of which the senses alone are competent to take cognizance. But all that is manifest to the senses in an act of causation is, as we have seen, insufficient to account for the existence of the notions whose origin we are in quest of. But it is an undoubted fact that all men, implicitly or dogmatically, hold certain connexions to be absolutely necessary and universal, as that two added to two must always make four.

We could not wish for a better specimen of the mind's being compelled to entertain the notion of necessity than Mr. Lewes exhibits, in his own case, in his criticism on "Hume's Theory of Causation," and on the "Fundamental Principles of Kant." While he labours hard to prove that there is no origin of ideas but what is denoted by the term "experience," he is all the while reminding us of Zeno in motion, when he denied the possibility of motion. Mr. Lewes maintains that there is something more in causation than antecedence and consequence, there is a causal nexus.

"It must be maintained that between those two events (cause and effect) there is a specific relation, a something which makes the one succeed the other, causing this particular effect rather than another; and this subtle link it is which is the nexus contended for; this relation it is which distinguishes a causal act from one of accidental sequence."

And "this subtle link," our author says, is apprehended by experience. Now here Mr. Lewes is actually preparing for taking his flight out of the confined nest of sensationalism, and does rise out of it a little, although he is careful to drop into it again. Mr. Lewes says that we have a tendency to think that two parallel lines will meet at some remote point, but that we correct this tendency by recurring to our experience. Now experience, which only enables us to obtain an indefinite notion of connexion, permits such a tendency, and when Mr. Lewes says that it is to be corrected by recurring to our experience, he is implying the faculty that does perform this office. Mr. Lewes affirms that all truths are necessarily true. If they are, it is assuredly not experience that informs us of the fact. Indeed the statement that all truths are necessary, is only another way of expressing what we have dwelt upon at a considerable length above, namely, that consciousness is veracious. Experience can only detect an indetermined conjunction between consciousness and veracity. The fact of our being constrained to confide in consciousness *as* veracious is no sufficient guarantee to reason that it is such: reason must know that the veracity we ascribe to it is *necessary* to the attainment of any certainty on this or any other point.*

* It may be observed that we have avoided the use of the phrase, "necessary truth," and substituted for it "necessary conjunction, or connexion of facts." If consciousness is veracious in its deliverances—*its demands being complied with*—all truths are necessarily true, not true to-day, but false to-morrow; but all conjunctions of facts are not necessary, for some are contingent. It is owing to this ambiguity in the phrase that we find Mr. Morell (Hist. of Modern Philosophy, 2nd Ed. vol. i. p. 298) stating that, according to sensational principles, "there can

Having attempted to prove that experience is not able to supply us with the notion of necessity, it may be thought that we claim for it an *a priori* origin—but we do not. We think, with Locke, that the mind is at first a *tabula rasa* in all respects, excepting, of course, its faculties; in short, that we possess no intuitions. We allow that such notions as goodness and badness, beauty and deformity, &c., have their origin in our emotional nature, but that the intellect has to discover what are the legitimate objects to which our emotions should be directed. For example, what is the true to the intellect, should be the good to the emotional nature; but to acquire the true there is no need of intuitions. We take our departure from the same point as Locke does, and follow the same method or way of transit, but we pursue it further than he does. We find within our mind notions which we cannot derive from our elementary faculties, at the same time we discover that without them they could not exist; and the manner in which we are conscious of obtaining them is this:—The elementary faculties provide the data: reason perceives the conclusion, which is a new truth, wholly distinct from any truth expressed by the data individually. Certain elementary cognitions *plus* the faculty of reason necessitate what otherwise could not possibly be known; and although we trace the dependence of the inferred truth upon the premises we can no more detect it in them severally (as we detect the particular in the universal) than we can detect water in oxygen and hydrogen. This intellectual procedure we call inductive reasoning.

It is commonly supposed that all reasoning is deductive, and since this kind of reasoning when conclusive starts from universals, that these are not established by reasoning. "Consequents cannot by an infinite regress be evolved out of antecedents which are themselves only consequents. Demonstration, if proof be no such thing as truth which may not at some time prove error." That rain fell to-day can never by any possibility turn out to be an untruth. What, then, does Mr. Morell mean? He evidently intends to express, that according to sensational principles, there are no facts conjoined which may not at some time be disjoined. The same ambiguity has led Mr. Lewes, in his strictures on Dr. Whewell's theory of necessity, to state—"I conceive that no such distinction whatever can be made out between truths which are necessary and truths which are contingent. All truth is necessary truth." If we substitute the word conjunction for the word truth in this quotation, it will be easily perceived that Mr. Lewes misapprehends Whewell's doctrine, and confounds necessary and contingent connexion between facts with a proposition's being clearly ascertained to be true, or not yet positively ascertained to be true, and so possibly untrue. The possibility of our being in error is a very different thing indeed from contingency in the connexion of facts, for about the existence on every hand of such contingency there is no mistake: every conjunction which is not necessary is contingent—thus there is a connexion between the word man and a certain being; take away the word man, and that being still exists: therefore the connexion between them is contingent. Contingency therefore equals non-necessity.

be possible, behoves to repose at last on propositions which carrying their own evidence, necessitate their own admission." True, but the primary data need not be necessary and universal. The premises of inductive reasoning are particular propositions, or facts of mere observation. We hold, then, that induction precedes deduction. Reason pre-eminently we regard as that faculty by which we obtain universals for the purpose of scientific deduction ; and without such universals we cannot conceive how we can arrive at other than probable conclusions.

Now having so far made ready the way for a formal enouncement of the process of inductive reasoning, we shall state what, after long, severe, and impartial testing, we deem the formula of such reasoning. But here we would call upon the reader to reflect that this formula is likely to be correct, exactly in proportion as it has already won a partial recognition ; and that if we had to propound a principle which had previously gained no amount of acceptance, we might be certain that it was a mere invention of our own ; for such truths do not grow up at once, like ephemeral insects, but slowly and for ages, like the oak. The formula is that implied by Sir John Herschel in his first two rules for finding out causes, that is, between them ; but more nearly approached to by Mr. J. S. Mill in his second Canon, and called the Method of Difference. Our contribution to this principle will be perceived, if it be not already perceived, as we advance. The way in which we obtain the formula is this :—We perceive a connexion between A and B : of the intrinsic nature of this we have at first no knowledge, and so relatively to ourselves call it indetermined or indefinite connexion ; and mere experience we contend cannot enlarge our knowledge in this respect. But we observe that if A be removed, B disappears. In this again, all that is obvious to experience is, that one event follows another, which, it has been ably contested, is no proof of causation. How then do we obtain such a notion ? By comparing together,

A is connected with B,
and
When this A is not, this B is not :

which being done, reason perceives that A is necessary to the existence of B. Now, without these premises, and a faculty to draw a conclusion from them, we cannot conceive that the human mind could ever be in possession of such notions as causation, necessity, dependence, essentiality, &c.

This formula admits of two variations, as follows :—

1st. A *plus* B,
*Minus* this A *minus* this B.
Therefore A is the cause of B.

2nd. *Minus* A *minus* B,
Produce this A you produce this B,
Therefore A is the cause of B.*

The *first* is the form of reasoning when we observe the supposed cause or causes, or presupposed entity in connexion with the effect or supposing or involving entity, and ascertain the negative premise in order to prove our previous belief. The *second*, which is substantially the same as the first (for we only start with the negative instead of the positive premise) is the form of reasoning when we search for effects instead of causes.

Time and space will not permit us now to point out how the premises of inductive reasoning are to be arrived at when the cause is concealed, as it generally is, by unessential concomitants; how we may know what is *not* the cause; and what affords *primâ facie* evidence of being such, before we obtain all that is requisite to prove it to be the cause; and how the cause is frequently to be ascertained by deduction from the universal, every change has a cause or its equivalent—all that cannot be the cause being previously abstracted.

We have now shown how necessary conjunctions are apprehended: how are these converted into universal propositions? Curiously enough this remaining part is accomplished according to the law of contradiction, which would be rightly termed universalization. For example, when we have ascertained that arsenic causes death by an induction in the second form, thus, before this man took arsenic he was quite well, when he took

* By A is to be understood whatever is necessary to produce B; and we believe that an effect is produced by the junction of two elements at least, as *one* added to *one* makes *two;* and that one of the elements *at least* must be modified by the other: they may modify *each other*, and that to such a degree as to become completely changed, as in chemical combinations. The word *this* in the second premise of these forms is to insure against a different A and B being meant in the second premise from what are contained in the first: thus, *this* man is a good mathematician, and he is a good reasoner: *that* man is not a mathematician, and he is not a good reasoner: therefore, &c., is a fallacious induction, because the negative premise is not negative to the positive, but is quite independent of it. The judge, who Mr. Macaulay relates, "was in the habit of jocosely propounding, after dinner, a theory, that the cause of the prevalence of Jacobinism was the practice of bearing three names," would have lost his fun had he been aware of this law. Perhaps the best way of stating the formula would be thus:—

1st. This A is B and it is C.
The same A is not B and it is not C.
Therefore B is the cause of C.

2nd. This A is not B and it is not C.
The same A is B and it is C.
Therefore B is the cause of C.

Here we regard B and C as attributes of the same substratum A: in the text they are regarded, under the titles of A and B, as abstracted from any substratum. The latter plan allows the formula to be stated more neatly than the former, which nevertheless answered more fully the purposes of our exposition.

it he died, &c., we also conclude that arsenic would kill any one to whom it was administered in sufficient quantity, and afterwards deduce from this universal. But how do we become convinced that it must be universal? If we suppose that arsenic some time or other may not cause death, we are supposing that there is only a contingent connexion between its being taken and the event which follows its being taken; that is, in stating the supposition we are forced to predicate contingency or non-necessity of necessity—*supposed* contingency of *demonstrated* necessity, which clearly shows that the supposition is not tenable. A triangle is a figure which *must* have three sides (the *must* would be here *implicitly* inferred), but a triangle may not always have three sides, are propositions which cannot both be true, but the first is *proved* true; consequently it is true beyond dispute that *all* triangles have three sides.

Here we must be looking out for the end; but before we conclude, it behoves us to inform the reader that, although we firmly believe that every man reasons inductively according to the formula we have propounded, we do not mean to assert that valid inductive reasoning always supposes the knowledge of it. We reason in accordance with it implicitly, long before we do so explicitly. Every mental operation takes place spontaneously before we become aware of its character. The knowledge of an intellectual process supposes that process, as the science of optics supposes the existence of sight. So far, we think, that Mr. Macaulay is correct in saying "that the inductive method has been practised from the beginning of the world by every human being.* But we cannot agree with him when he says, "We think it is quite possible to lay down accurate rules . . . . for the performing of that part of the inductive process which all men perform alike, but that these rules, though accurate, are not wanted, because in truth they only tell us what we are all doing." We must lay particular emphasis on the fact that implicit induction only sufficed for establishing the data of our earliest deductions,† and those of the elementary sciences. When data

* Essay on Bacon.

† It is surprising how different facts are made to appear according to the theory which is brought to bear upon them. Mr. Mill (System of Logic, &c. i. p. 210) writes:—"Not only may we reason from particulars to particulars without passing through generals, but we perpetually do so reason. All our earliest inferences are of this nature. From the first dawn of intelligence we draw inferences, but years elapse before we learn the use of general language. The child, who, having burnt his fingers, avoids to thrust them again into the fire, has reasoned or inferred, though he has never thought of the general maxim—fire burns. He knows from memory that he has been burnt, and on this evidence believes when he sees a candle, that if he puts his finger into the flame of it, he will be burnt again. He believes this in every case which happens to arise; but without looking in each instance, beyond the present case. He is not generalizing; he is inferring a particular from pertaining to subjects of a more advanced and less accessible and intelligible nature had to be obtained, the implicit process failed. While men were scientific in geometry, they were romancers as to the stars; and now that they have won its secret from the heavens, and can sail round the world by the light of science, they still trust to empiricism for navigating the ship of state. When facts were obtruded upon the mind, and were possessed, so to speak, of a perfectly transparent aspect, or admitted of being painted on the imagination with the vividness of reality, universals would follow as naturally from them as talking from the possession of the faculty of language and a vocal apparatus. But where facts did not come unsought into the mind, and when they were not distinctly and clearly possessed, there would be no implicit induction. For unless the premises of inductive reasoning had that character which, in concert with reason, constitutes them causes of universals, no universals would follow. But for the premises to be of this lucid nature, and for us to be aware of the form they should assume, are two very different things, as different as trusting entirely to nature for the supply of our wants, and supplying them artificially. And thus it is that we possess some data so fully, but cannot account for their genesis, and consequently call them *self*-evident. But there are some who, in attempting to account for their origin, deny their universality, and call them mere generalizations from experience. There is truth on both sides, but error also. Truth is generally brought to light by conflict between those who ask too much and those who grant too little—between the dogmatists and empirics on the one side, and the sceptics or critics on the other. Men, however, must begin by being dogmatists; they must learn the alphabet of science, and spell out its easier parts before they become proficient enough to undertake more recondite researches; and their reasoning at first would consist in drawing conclusions from universals implicitly obtained. This method of reasoning deductively from data spontaneously supplied would naturally be imitated wherever it was found that unaided efforts were not equal to the task of clearing up the mysteries of being. But its use would be limited in such cases to the knowledge they had of it, to the extent to which it had become explicit. Axioms particulars." Mr. Mill here very clearly describes what is *explicit* in our earliest inferences; but what really does take place in the unexplored recesses of the mind we believe to be that process which we have attempted to explain—namely, to take the example given by Mr. Mill, an induction in the second form, thus:—Before the child touches the fire he is *not* burnt: but when he does touch it he is burnt; therefore the child concludes *implicitly* that touching the fire is the cause of his being burnt: which conclusion he *implicitly universalizes* into All fire burns: from which universal he ever afterwards *implicitly* deduces, that if he puts his finger in the fire it is sure to be burnt.

and definitions being considered *self*-evident, attention would be centred, as a matter of course, on deduction, hence the development of this portion of reasoning long before the other, and the almost universal application of the deductive method to all branches of knowledge. Where implicit induction accomplished all that was needed, several sciences were established with small reflective knowledge of the processes of reasoning; but where this spontaneous procedure was checked by increasing turbidness and depth in the widening river of knowledge, men endeavoured to supply its place as best they could, and this would be at first by a very crude imitation. In room of universals supplied spontaneously by Nature's bounty, they would invent what they thought the most reasonable principles, imagining, as they at first could not avoid doing, that all first principles must be educed from the mind. The consequence was "the multiplication of systems in every conceivable aberration from the unity of truth," teaching men the salutary lesson that they must learn before they can teach. Bacon rightly describes this era in reasoning, it seems to us, therefore, when he says, that men have sought to make a world of their own conceptions, and to draw from their own minds all the materials which they employed. It was inevitable that they should; this was the provisional step which prepared the way for the scientific period. It was imitating the model they possessed as far as they understood it. But even this primitive method, when applied to less accessible phenomena, involved some degree of observation, for without that it would be impossible to invent a theory bearing any relation to the facts; and thus would be partly laid the foundation of the explicit development of inductive reasoning.

But a great forerunner of scientific induction was that spontaneous generalization from experience, which resulted from man's being placed amidst those numerous uniformities of nature which every moment of his waking life could not otherwise than attract his notice. These spontaneous generalizations would be so many data from which men could reason deductively. But how many of them would be legitimate data, and how many not? Such of them as were simple enough to permit of implicit induction taking place would be established universals; but such of them as did not admit this would be no better than indetermined conjunctions, and any conclusions drawn from them might be true—might be not. Now it was only in the former of these instances—that in which implicit induction took place, that the idea of causation would be arrived at. In the latter, where *inductio per enumerationem simplicem* was all that occurred, the idea of causation was unattainable, for here only one of the premises of inductive reasoning was to be met with: whereas both are essential to our acquiring the notion of one thing being necessary to the existence of another. When Mr. J. S. Mill, therefore states that,

> "As all rigorous processes of induction presuppose the general uniformity, our knowledge of the particular uniformities from which it was first inferred was not of course derived from rigid induction, but from the loose and uncertain mode of induction *per enumerationem simplicem:* and the law of universal causation, being collected from results so obtained, cannot itself rest on any better foundation."*

When he states this he is clearly in error: causation can only be inferred from *rigid* induction; and the law of causation is an universal, which the mind cannot help acquiring by implicitly abstracting from *specific* universals spontaneously obtained *that* in which they all agree, thus resting in the *generic* universal—the axiom of induction, namely, Every thing which does not exist *per se* exists *per aliud;* that is, it is either primordial and presupposes nothing, or it is not primordial and supposes an antecedent.

But in the latter of the instances mentioned above, namely, where merely *inductio per ènumerationem simplicem* took place, many of the principles of physical science would be gained, because this loose and unscientific method would be applied in cases where the use of the complete method would have been rewarded with successful results. As objects requiring the use of the inductive method came to be seriously inquired after, in the same degree there grew a demand for its further explicit developement. So while the study of natural philosophy created a demand for the inductive method—that method by this means, first empirically, and then scientifically, disclosing itself would facilitate the solution of more abstruse problems, by turning mere forest tracts into broad and level roads, and superseding the picturesque, but unsafe, stepping-stones of a ruder period by bridges finely constructed.

Taking the view that we do of the value of a clear knowledge of the laws of reasoning, the question—what is the use of logic? is easily answered by replying, that it cannot be dispensed with. When this question has had to be answered by some of our most eminent logicians, after they had made the admission that logic could merely tell us what we were all doing admirably well already, it required no small amount of ingenuity and enthusiasm to plead on behalf of the study of this science, for there were persons always ready to object that the knowledge of the processes of reasoning aids us no more in the practice of it than an acquaintance with anatomy enables a man to walk any better

* System of Logic, ii. p. 97.

than one profoundly ignorant of that science. But this remark applies solely to *implicit* reasoning, which we have shown does not go with us much beyond the confines of the region we have to explore. That we need an explicit statement of the laws of thought from first to last, let the battle that is raging between Positivists, Individualists, and Traditionalists, assure us. We feel convinced that nothing but the general recognition of the One True Method will dispel the anarchy which now exists in matters of a social and moral kind. Because we can reason implicitly on questions demanding little exertion of intellect to comprehend them, and indeed on abstruser subjects when we devote so much attention to them as to make them thoroughly our own,* it does not follow that we can reason on all sorts of intricate questions—beheld, too, through the obscuring Gehenna atmosphere of self-interest and prejudice, any more than the earth is able of itself to supply all that an advanced agricultural skill can win from it, because in the first place it yielded sustenance spontaneously to its rational inhabitant—man. We think that the minds of individual men (none of them harmoniously developed, but full here, defective there) in exploring the vast unknown need help; and that this help is to be derived from a reflective knowledge of the mind's own laws, those fundamental facts, varying in degree, but never in kind, which underlie the more variable attributes of thought, and which cannot be absent without the mind's being dethroned thereby. The individual mind is not self-sufficient: it must be consciously endeavouring to obey the Laws of human thought. Bacon, says Coleridge, "supposes that the Intellect of the individual or *homme particulier*, may be refined by the Intellect of the Ideal Man, or *homme général*."† And it is superfluous to state who it was that said—"The real cause and root of almost all the evils in science is this, *that falsely magnifying and extolling the powers of the mind*, we seek not its real helps."

> "Though various foes against the truth combine,
> Pride, above all, opposes her design."

---

* This is what Dr. Whewell seems to have in his mind when he says, "I must explain, that I do not by any means assert that those truths which I regard as necessary are all equally evident to common thinkers, or *evident to persons in all stages of intellectual development*. I may even say, that some of those truths which I regard as necessary, and the necessity of which I believe the human mind to be capable of seeing, *by due preparation and thought*, are still such, that this amount of preparation is rare and peculiar; and I will willingly grant, that to attain to and preserve such a clearness and subtlety of mind as this intuition requires *is a task of no ordinary difficulty and labour*."—Letter to the Author of "The Prolegomena Logica."

† Treatise on Method, sect. ii. p. 52. Encyc. Met.

